# The key therapeutic factors needed to deliver behavioural change interventions to decrease risky substance use (drug and alcohol) for looked after children and care leavers: a qualitative exploration with young people, carers and front line workers

**DOI:** 10.1186/s12874-019-0674-3

**Published:** 2019-02-21

**Authors:** Hayley Alderson, Rebecca Brown, Alex Copello, Eileen Kaner, Gillian Tober, Raghu Lingam, Ruth McGovern

**Affiliations:** 1 Institute of Health and Society, Newcastle University, Newcastle Upton Tyne, UK; 2 School of Psychology, University of Birmingham, Birmingham, UK; 3 Leeds Addiction Unit, 19 Springfield Mount, Leeds, UK; 4 Faculty of Medicine, University of New South Wales, Sydney, Australia

**Keywords:** Looked after children, Drugs and alcohol, Social work, Intervention

## Abstract

**Background:**

Looked after children and care leavers have an increased risk of drug and alcohol use compared to their non-LAC peers. Despite high prevalence rates within this population, looked after children are reported to show low levels of engagement in services resulting in unmet needs emerging from substance use. This paper reports on the initial formative phase of a pilot feasibility randomised controlled trial; SOLID (Supporting Looked After Children and Care Leavers In Decreasing Drugs, and Alcohol) that aimed to adapt two evidence-based psychosocial interventions, Motivational Enhancement Therapy and Social Behaviour and Network Therapy, which will aim to reduce substance misuse by looked after children.

**Methods:**

We conducted qualitative semi-structured interviews and focus groups with 19 looked after children aged 12 to 20 years old, 16 carers and 14 professionals across four local authorities in the North East of England. The data gathered were analysed and then presented within co-production workshops inclusive of 13 young people and 14 professionals (drug and alcohol practitioners and social workers). Findings were used to adapt and refine the interventions prior to the trial.

**Results:**

Overall findings suggested that whilst original components of both interventions were feasible to deliver and acceptable, specific process areas were highlighted including: increased emphasis upon therapeutic relationships, the benefits of using creative non-traditional methods of engagement and identification of treatment goals wider than those narrowly focused on substance misuse.

**Conclusion:**

This paper provides an example of methods used to collect multiple perspectives to refine and co-develop interventions to reduce drug and alcohol use in the specific population of looked after children.

**Trial registration:**

ISRCTN80786829 (first registered 06.06.2016- prospectively registered).

## Background

One in every 156 children in the UK is looked after by the state [1]. These looked after children and care leavers (henceforth denoted as LAC in this text) have often experienced multiple forms of abuse and neglect. They have an increased risk of drug and alcohol use compared to their non-LAC peers. For example, 25% of LAC in foster care and 42% of LAC in residential care drank alcohol at least once a month, compared to 9% of young people not looked after [2]. A national survey of care leavers showed that 32% smoked marijuana daily [3] compared to a recent estimate of 5% of young people aged 16–24 years in the general population [4] and data from 2012 showed 11.3% of LAC aged 16–19 years had a diagnosed substance use problem [5]. In addition, LAC have almost fivefold increased odds of at least one mental health diagnosis compared to their non-LAC peers (disorders including anxiety, depression or behavioural disorders) (OR: 4.92; 95% CI: 4.13, 5.85), further increasing their risk of substance use and poor life chances [6].

LAC have often experienced family breakdown, neglect and abuse prior to entering care and have had little or no control in many aspects of their lives including their living arrangements or ‘placements’. This level of disruption often contributes to distrust of and lack of engagement with professional services [7], resulting in the needs of LAC often being unmet. The National Institute for Health and Care Excellence (NICE) [8] and the Chief Medical Officer specifically highlighted LAC as a vulnerable group of young people who needed effective interventions to reduce their risky drug and alcohol use [9]. However, there is a lack of research adapting interventions for use with LAC and assessing their effectiveness at scale.

This paper provides a case study for developing interventions with this ‘hard to reach’ group of young people, it draws on their views and lived experiences to ensure the interventions are salient. We aimed to establish whether the Motivational Enhancement Therapy (MET) and Social Behaviour and Network Therapy (SBNT) interventions were feasible and acceptable to adapt in relation to LAC and other key stakeholders. The adaptation process occurred through a series of stages, which were all qualitative in nature. The steps taken are documented and we present qualitative research findings that illustrate how insights from LAC and care providers influenced the adaptation and manual development of our two evidence based interventions to reduce drug and alcohol use and to be delivered in mainstream alcohol and drug treatment services.

## Methods

### Step 1: Selecting interventions for adaptation

Motivational Enhancement Therapy (MET) and Social Behaviour and Network Therapy (SBNT) are existing interventions that have been shown to be effective in decreasing substance use in a range of participants including adolescents [10–12]. MET is a client centred, directive counselling approach developed as a concentrated version of motivational interviewing (MI) [13]. The basic assumption of MET is that the motivation and responsibility for change lie within the client, and it is the therapist’s role to create an environment to enable the client to change. A review by Carney and Myers (2012) has shown that MI and MET have shown promise for adolescents with problematic substance use [14–16].

SBNT is a systematic counselling approach, which utilises cognitive and behavioural strategies to help clients build social networks supportive of positive behaviour change in relation to their substance use and goal attainment [17]. LAC are often engaged in substance misusing social networks, which reinforce their risk taking behaviours. NICE have recommended family interventions when working with young people presenting with complex needs such as substance misuse and mental health [8]. However, for LAC, it is unlikely that the more traditional family interventions would be feasible given the potential for family fragmentation and broken relationships within this complex group. As such, SBNT offers an alternative intervention with potential to galvanise a supportive network for the child that can also draw from social support beyond the immediate family.

However, whilst both MET and SBNT have been shown to be effective at reducing substance use, less is known about their effect with young people who are looked after by the Local Authority and likely to have more fragmented family relationships. The nature of social networks and how they differ for LAC within the looked after system is paramount to understanding how to effectively engage and work with this group of young people.

### Step 2: Developing a theory of change model

In accordance with guidance from the Medical Research Council (MRC) on developing complex interventions [18], we commenced the adaptation of the interventions by building a theory of change model relating to our target population. The central vulnerabilities of LAC, is the absence of appropriate family support and supervision and the life experiences which led to their placement within care. Based on existing theory we predicted that an intervention seeking to decrease substance misuse by this group would need to address these wider social and environmental determinants. We developed Behaviour Determinants Intervention (BDI) models for each intervention, to identify how the research team visualised the proposed change pathways for the interventions, highlighting the determinants for change and key behaviours targeted by the intervention.

Within MET, the therapist could employ strategies to build and strengthen motivation by eliciting self-motivational statements from the LAC and that in doing so, the LAC could resolve the inherent ambivalence about their substance misusing behaviour [10]. When developing the BDI model we thought MET would strengthen motivation but we also thought that it would be helpful to provide personalised feedback to assist the young person to consider risks and tip the decisional balance. We were also aware that risks and decisions may differ somewhat from those of adults e.g. they are more likely to be acute or shorter term such as friendship difficulties or regretted sex rather than abstract future orientated risks such as high blood pressure, to really explore the reasons for change.

Within SBNT, the therapist could employ cognitive and behavioural strategies to help LAC build social networks to help shape positive behaviour change in relation to their goal attainment [17]. When developing the BDI model we thought SBNT could encourage LAC to recognise potential broader, informal networks of support outside of traditional caregivers. Social network support is key in helping people deal with problem behaviours inclusive of substance misuse. We were aware that networks of support available to LAC would differ from children and young people residing within more traditional biological families.

**Fig. 1:**
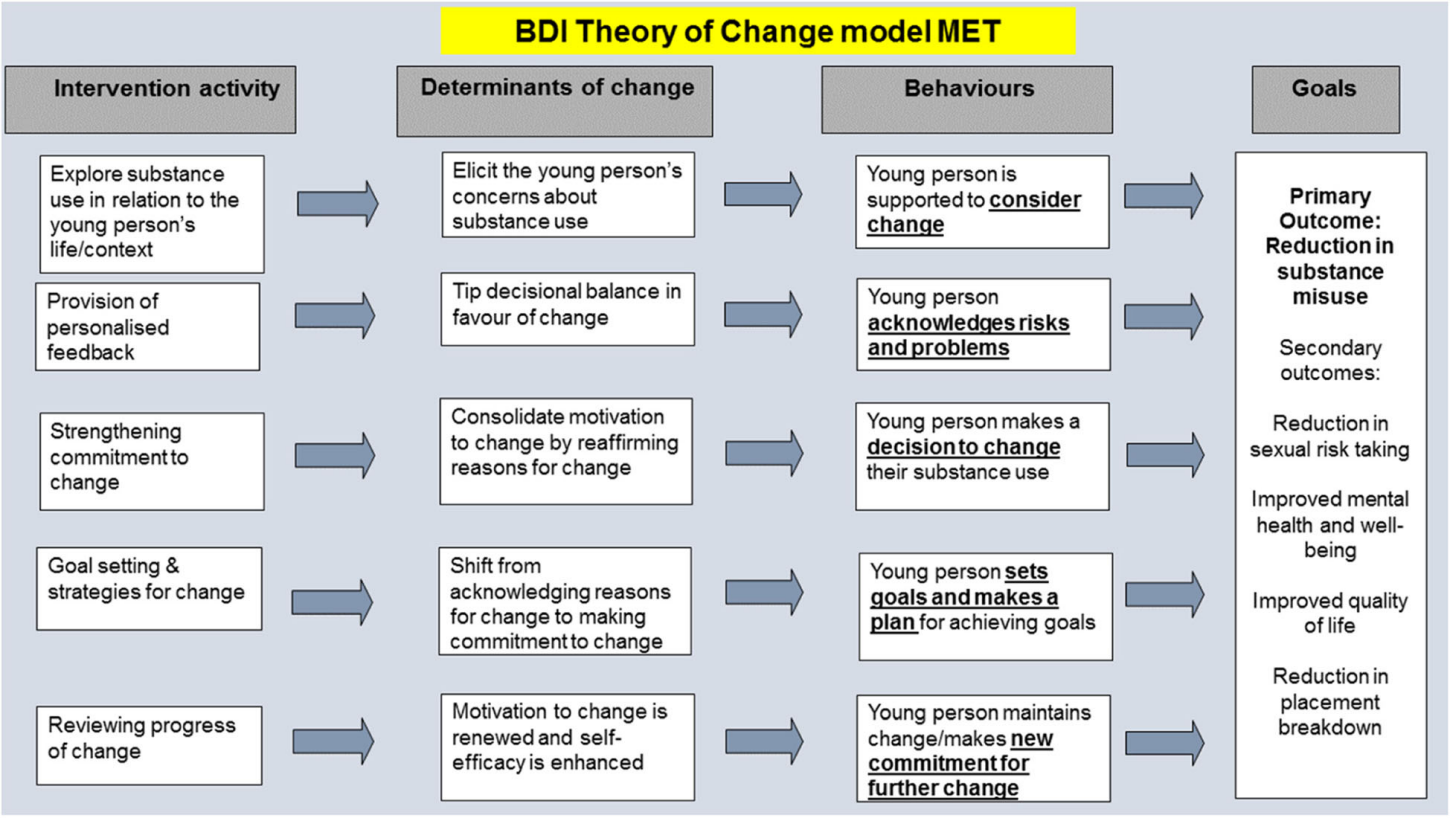
BDI Theory of change model

**Fig. 2:**
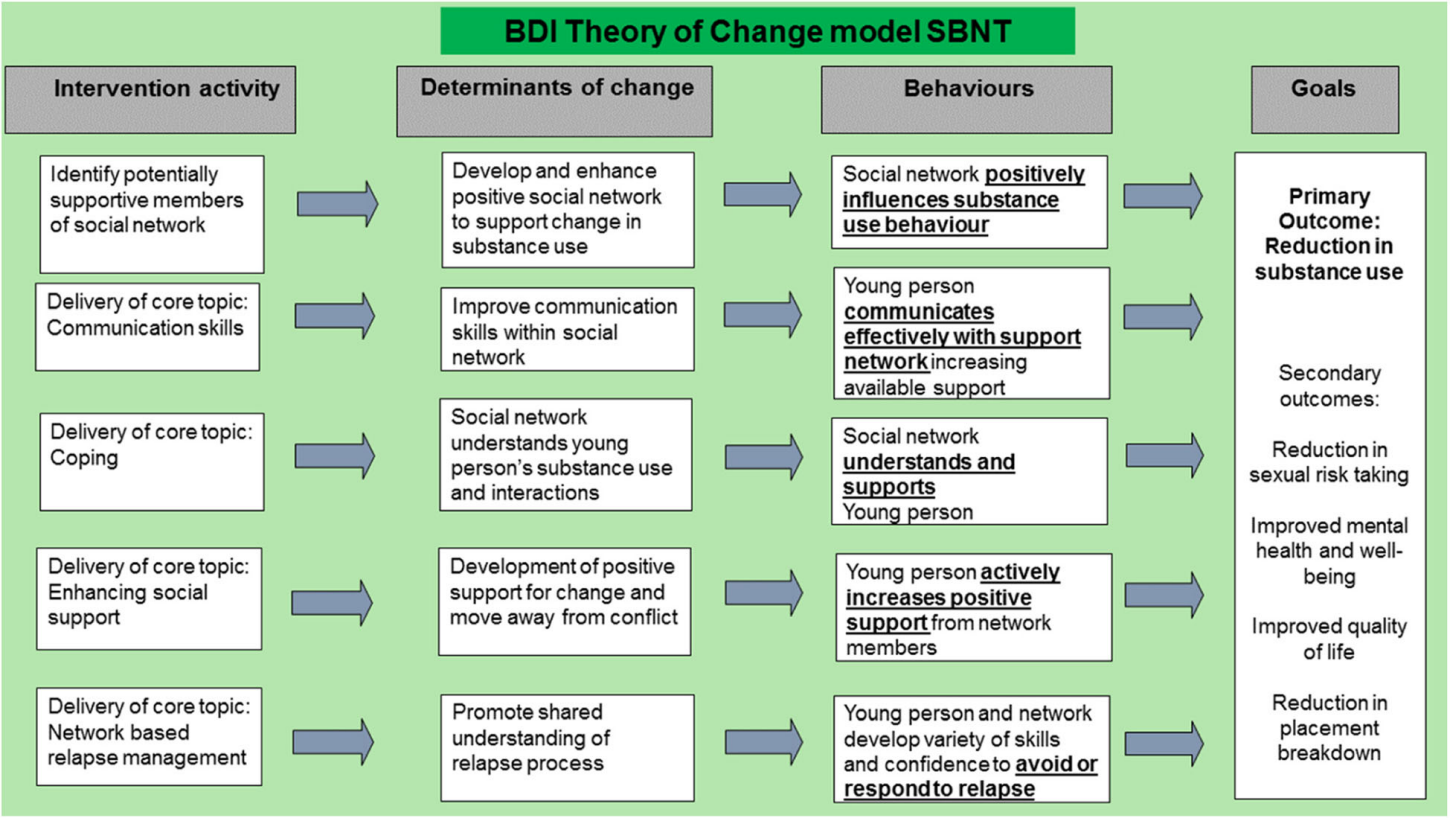
BDI Theory of change model SBNT

Semi structured topic guides were developed to discuss the assumptions inherent within the logic models (Figs. 1 and 2) and to explore the key behavioural and motivational domains that the interventions should address. Qualitative research was undertaken to increase our understanding of the needs of this group of LAC.

### Step 3: Qualitative study

#### Qualitative interviews and focus groups

We used qualitative methods to establish whether the MET and SBNT interventions were perceived as feasible and acceptable and to adapt them in relation to LAC and other key stakeholders. We proposed to carry out individual 1:1 interviews with LAC and carers and focus groups with professional participants. In reality, for pragmatic reasons we conducted a combination of individual interviews, dyad interviews and focus groups depending on participant’s availability. Table 1 shows the methods used to recruit participants and provides the demographics of the study participants involved in the first round of qualitative work.

**Table 1:** Qualitative participants

Qualitative method	Participant group	Number of participants	Gender	Placement type/job role	Substance use
Individual interviews	LAC	19	F = 9, M = 10	Foster care = 5 Residential care = 8 Independent/supported living = 5 Living with biological parent = 1	Current/previous substance use = 16 Never used substances = 3
	Carers	13	F = 8, M = 5	Foster carers = 6 Residential workers = 4 Supported living workers = 2 Biological parent = 1	
	Drug and alcohol workers	3	F = 1, M = 2	Service manager = 1 Drug and alcohol worker = 2	
Dyad interviews	Social workers	4	F = 4	LAC managers = 2 Social workers = 2	
Focus groups	Drug and alcohol workers	5	F = 3, M = 2	Service manager = 1 Drug and alcohol worker = 4	
	Social Workers	4	F = 3, M = 1	Senior Social workers = 3 Social worker = 1	
	Carers	4	F = 3, M = 1	Foster carers = 4	
Total participants		52			

Qualitative interviews were carried out with a purposive sample of 19 LAC. The LAC were identified by their social workers within each local authority area, who acted as the gatekeeper. Social workers provided the research team with contact details for LAC whom had agreed to be contacted. The research team then contacted eligible participants (LAC aged 12–20 years, screened positive for being at risk of substance use, able to provide informed consent and residing in the study area) to discuss the research and arrange an appropriate time to visit and conduct an interview. The purposive sample aimed to ensure diversity with regards to age, exposure to drug and alcohol use and placement type. The final sample was representative of the LAC population so far as there was an equal mix of male and female participants and a range of placement types across the different local authority areas as identified in Table 1. Sixteen carers, inclusive of a mixture of foster carers and residential children’s home workers supporting LAC were involved in 13 individual interviews and a focus group with 3 participants. The carers were identified by the social worker to ensure diversity of sample in terms of carer type (i.e. foster carer/ family member/ residential worker), the participant demographics are identified in Table 1. Furthermore, it is important to acknowledge that the carers were a mixture of those caring for LAC interviewed as part of the study and those who were recruited for an interview independent to a LAC. Interviews were the preferred method used with LAC and carers (foster and residential) as interviews covered sensitive issues and explored personal experiences of drug and alcohol use, barriers and facilitators to engaging with services and any recommendations for service improvements. The focus group with carers emerged incidentally and for pragmatic reasons as one carer was part of an already established support group that we were invited along to, 3 members of that group agreed to participate in a group interview.

Interviews were also carried out with 3 drug and alcohol workers whom had previously delivered SBNT as part of a previous trial [12]. These interviews aimed to build upon previous knowledge and experience of delivering MET and SBNT to young people and contribute towards adapting the treatment manual for the LAC population.

Focus groups and dyadic interviews were held with professionals. Two focus groups were held, one with 4 LAC social workers and one with 6 specialist young people’s drug and alcohol workers. This was complimented with two further dyad interviews with social work staff. The focus groups and dyad interviews enabled us to discuss the original components and principles behind the MET and SBNT interventions and whether they perceived them to be relevant to LAC. They also enabled us to explore the broader therapeutic approaches required to work with LAC, the feasibility of delivering the interventions to LAC and consider potential barriers to delivering the interventions at scale. Data were collected until data saturation was reached.

Written informed consent was obtained for all participants, inclusive of assent from LAC under 16 years of age and consent from their corporate guardian. Information regarding the consent procedure and interview process can be found in the SOLID protocol paper [19]. Interviews were carried out by experienced qualitative researchers, they were audio recorded and transcribed verbatim. Transcripts were anonymised and identifiable participant details with a participant key were stored separately. Pseudonyms are used within this paper to maintain participants’ anonymity.

### Step 4: Qualitative analysis and findings

A preliminary thematic analysis was conducted on the data available from the qualitative interviews and focus groups, in preparation for the intervention development workshops with professionals and young people before finalising the adapted MET and SBNT manuals.

Transcripts were thematically analysed [20], in practice this entailed a line by line coding process and then analysis within a given transcripts and across the dataset as a whole. Analysis was an iterative process, using the constant comparative method [21], in order to identify key themes and concepts. Qualitative software (NVIVO 10) aided the organisation of thematic codes. The data were compared across the three participant groups (i.e. LAC, professionals and carers) with similarities and differences being highlighted. Further, we compared and contrasted the main themes within the BDI models described above in order to challenge the existing models in order to refine and adapt the existing versions of the interventions. In the first instance, data were analysed by both RB and HA in order to ensure inter-coder consistency and agreement. The main themes and findings were presented to the wider multidisciplinary team (RL, RM, AC, GT, and EK) whom have expertise in a variety of backgrounds including: public health, social care, social science, drug and alcohol use, and clinical psychology. HA and RB then finalised the themes and main findings in order to develop recommendations for the adaptation interventions. Again, these were presented to the broader team for discussion in preparation for the intervention development workshops which are described in more detail below.

The quotes included in this paper came from young people who have experienced receiving drug and alcohol treatment interventions and/or LAC accessing other services for support surrounding ‘help seeking’ behaviour. Additionally, opinions were sought from professionals who have experienced delivering interventions and engaging with the LAC population.

### Key themes and how they influence manual adaptation

At the outset of this attempt to co-produce and adapt two distinct intervention manuals for SBNT and MET, topic guides were written to focus on each intervention separately and in its own right. What became apparent as an emergent finding is that less emphasis was placed on the specific intervention being delivered and more importance was placed on the ‘key therapeutic factors’ and generic processes of working with LAC. Therefore, the main findings focused on ‘therapeutic relationships’ and ‘engagement and challenges of working with LAC’, which are relevant regardless of the intervention being delivered.

The themes and subthemes that emerged from the data within each of these categories are described below and the discussion section outlines how the data collection influenced our manual and training adaptation.

#### Theme 1: Therapeutic relationships

The qualities of trust and genuine care were the two main sub- themes that emerged regarding what underpinned a successful therapeutic relationship. Participants, inclusive of professionals and LAC themselves highlighted the importance of building a therapeutic relationship when working to reduce substance misuse. The LAC’s ability to confide in professionals and trust the substance misuse practitioner was a recurrent theme. Whilst trust is recognised as a necessary condition for any caring relationship, it was reported to be particularly important for LAC, whose experiences leading up to their placement in care may have impacted upon their ability to trust others. Professionals acknowledged that LAC often experience disorganised and difficult attachment. This included repeated experiences of their essential needs going unmet, relationship breakdown and abandonment, being let down and broken promises. Professionals displayed a clear understanding of these complex attachment issues and discussed the need to ‘earn’ trust when engaging with LAC;

*“You need to put in the groundwork initially. I think with teenagers you need to gain their trust, you need to work for it. Because if they have been hurt, which they will have been, they will try to push you away. They won’t want to trust you.” (Carly, Social worker, focus group)*

Further, LAC described seeking qualities such as reliability, empathy and partiality, which may have been qualities missing in their early attachments. Practitioners were expected to act in particular ways in order to demonstrate their trustworthiness. Typically this involved the practitioner being reliable; a quality which practitioners reported could be communicated to the LAC in multiple ways within the interaction. One foster carer describes displaying their reliability in terms of being available 24/7, he is permanently ‘on call’ if a young person needs him, he states;

“*it is not a job because there is no job that makes you work 24 hours a day, 7 days a week and 365 days of the year, but this one does” (James, Foster carer, focus group).*

Professional and LAC participants reported that the practitioner’s reliability must be consistent as any inconsistency is likely to build mistrust.

*“Just by keeping to your word, even little things like keeping your appointments and attending on time, looking into things when you say you’re going to…” (Susan, Social Worker, focus group).*

From the perspective of LAC, engaging with services depends fundamentally on the relationship between themselves and their allocated worker. To facilitate the sense of a reciprocal trustworthy relationship, young people explained the importance of ‘working gradually’, wherein at least the first couple of interactions should be dedicated to building a rapport and ‘engaging’ the young person prior to formal sessions commencing. Additionally, this could be shown by professionals not expecting young people to instantly make disclosures, but allowing a positive working relationship to develop first. Self-disclosures where practitioners ‘trade’ personal information were perceived to be beneficial to developing a trusting relationship, whereby the process of sharing information was not completely one sided. Some, examples that young people provided for this were discussing a hobby that the practitioner enjoyed doing or talking about a pet they had. This level of disclosure enable a small ‘trade’ of personal information to be made without divulging any sensitive personal information. LAC reported that such disclosure enhanced their sense of connection to the practitioner as well as their own safety to disclose information.

*“When you work with someone you have to build a bond up first, before you can open up to them…..It’s, well the way I’ve done is just ask questions about them, and then if they tell you, then you know well if they’ve told me this then I can tell them that” (Sophie,17, YP interview).*

A further quality that LAC sought but did not always feel that they received was that of ‘genuine care’. LAC described having multiple contacts with professionals, with much of the care a child usually receives from a loving family being provided by a professional who is employed to provide such care. The corporate parenting role dictates that safeguarding and risk management take precedent over the provision of emotional support. However, many social workers described going ‘above and beyond’ their role and being available outside of their contracted working hours in an attempt to show they care for the young people in their care.

*“Myself and his YOT worker had agreed between us that we would have our phones on 24/7. So that if he wanted to get in touch and check in we knew he was okay. So we did, we took turns and he did check in and he did arrange to meet up which was really good” (Steph, Social worker, focus group)*

LAC were acutely aware of the corporate parenting role fulfilled by the professionals and highlighted the importance of practitioners (professionals and foster carers) whom made them feel like they ‘genuinely’ cared about their welfare. Despite being in a paid position to provide care for young people, foster carers reinforced their attempts to provide the same level of care and support to the children and young people they foster/care for in the same way they would treat their own biological children.

*“Any child that comes to live with me, I know they are not mine, however I will work with them, I will play with them, I will live with them and I will do everything to my best ability in every area, in every arena because I want what is best for them.” (Liz, foster carer, interview).*

For LAC, Genuine care involves professionals ‘being available’ when needed, showing empathy, perseverance and providing support (emotional and practical) which feels unconditional. For the young people, genuine care was described as stemming from personal investment rather than a professional obligation or remuneration.

*“Like Josie talks to me, not like I’m just someone she has to work with, she talks to me like she cares” (Carla, 17, YP interview)*

From the perspective of LAC, a further way of professionals showing that they cared for a young person was to take a non-judgemental approach and to show unconditional positive regard to the young people under their care regardless of the information they were disclosing. This was reinforced by professionals and foster carers, whom reported LAC disclosing information to them regarding historical experiences. Foster carers described having to respond in a sensitive and non-judgemental way.

“*We had a young man who had been abused by a family member. He was feeling guilty himself about it and thought that we would feel disgusted that things like that had been done. It is letting him see that we are not disgusted. Straight away, I have heard all of this before, you are not the only one. It is not your fault.” (Carol, female, foster carer, focus group).*

This was important to LAC as some participants felt that they may be judged because of their experiences leading up to them being placed in care and elected to not engage for fear that practitioners would not be able to ‘cope’ with their experiences.

*“…my family is ‘f…. up’…really ‘f….. up’. And if I sat there and told someone they’d probably run a mile, they probably would. So that’s why I’ve never really opened up to anyone, cause if I did they probably would run away, do you know what I mean?” (Ewan, 17, YP interview)*

The above examples highlight the specific skills necessary to facilitate a therapeutic relationship. Both LAC and professional identified additional practical issues and challenges that needed to be address within the adapted manuals.

#### Theme 2: Engagement and challenges of working with looked after children

There was a consensus from all perspectives that traditional one-to-one counselling style interactions are often unproductive for LAC. Typically this was experienced as overly formal for LAC who might find this type of interaction difficult to engage with. Young people commented on how they found it harder to participate in ‘traditional’ formally structured sessions.

*“It was like in a room…and like there’s a table there and it had like little seats round, and like, he was just on about things. Do you know, he didn’t make it very good, like, he didn’t make it very fun and enjoyable kind of thing. It was just like, boring. He was just writing things down that I was saying basically and it just upset me. He just kept on going over it and over it and over it, he was like “so how did that feel? Bla bla bla.” I didn’t really feel comfortable” (Isabelle, 13, YP interview)*

The ability for practitioners to work creatively and use visual strategies such as the ‘node-link mapping’ used in the International Treatment Effectiveness Project (ITEP) and mood cards whilst staying true to the intervention delivery was deemed a successful strategy to engage LAC.

*“That are not many young people who you’ll get to the point where you’re doing that one to one counselling really. It is few and far between. You’re being creative…” (Adam, drug and alcohol worker, focus group).*

Many LAC wanted other strategies and approaches to be used to help them connect with professionals, maintain concentration and become more involved in sessions.

*“Writing it down or doing it like arts and crafts way because I don’t like just talking and having conversations cause I just get a bit bored and lose track, then I’ll start fiddling about.” (Abbie, 18, YP interview)*

A further approach deemed necessary when working with LAC was to explicitly acknowledge the complexities of their life due to them being in the care system. This enables a holistic approach to be taken within sessions. LAC identified it was important that goals did not focus solely around substance use. They valued discussions that recognised the difficulties occurring within their lives and facilitated a personalised approach to be taken to meet their needs. Professionals also clearly identified that a bespoke approach has to be taken;

*“I think what’s coming out here is that with the kids we work with, the drug and alcohol issue is over there, if you like, and a whole raft of other issues are here. As workers we’re dealing with all of these here and that tends to sort the drug and alcohol issues out quite naturally” (Laura, Drug and alcohol worker, focus group)*

Frequent placement changes resulted in inconsistent and fragmented support networks for LAC. The transient nature of the LAC population can result in young people being eager to find friends even if that results in becoming involved in unhealthy friendships.

*“So they might, you know, have contact with their brothers or sisters, you know, it is just they get moved around, and when they are moved around they are vulnerable, they are desperate to have friends or they are desperate to have somebody to call their own…… people get attracted to them who are, I would say, not the type of kids I would want my kids to knock around with” (Liz, foster carer, Interview).*

This was highlighted as a challenge when working with LAC. Whilst social support networks is an explicit component of SBNT, LAC and practitioners recognised the central part that social interaction and support for change plays in any resolution of substance misuse problems.

*“It is quite sad sometimes when they haven’t got anybody in the family, not even an uncle or a cousin or somebody who they can put down as a support really” (Steph, social worker, focus group).*

The challenges of finding appropriate network members was explored, in many interviews LAC struggled to identify someone they felt they could turn to, feelings of not having support or the need to be self-sufficient was verbalised;

*“My boyfriend and his friends, and there’s a few of my friends. Actually they’ve got their own lives as well, they’ve got their own houses and their partners and they’re all settling down as well, so…there’s not really many people there. When you think about it though, how many of them can you turn to if you’ve got a problem? Cause there’s not a lot” (Abbie, 18, YP interview).*

When young people did identify positive support, it was often people outside of the traditional family support network as would be expected within the LAC population. This in itself could be challenging due to the identified sources of support often being professionals whose ability to provide ongoing or out of hours support is not always practical as would be possible from a more traditional family member.

*“There’s two main people I’ve got in my life which provides me with support. One’s my boss, he’s a farm manager, I work with him most days. Another person is the manager of [name of school], he owns the company and he helps quite a lot by, when I moved out of here the first time, he’s the one that made me come back, and let me get my head back” (Philip, 17, YP interview).*

### Step 5: Co-production of intervention manuals

The preliminary findings from the qualitative interviews and focus groups were presented within a series of intervention finalisation workshops that took place to co-produce the final intervention manuals. Five workshops were conducted, one with professionals (*n* = 14) all of whom had been involved in step 3 of the process earlier. Four workshops took place with young people (*n* = 13), none of whom had been involved in the study previously, Table 2 provides the demographics of the study participants involved in the qualitative finalisation workshops.

**Table 2:** Qualitative Finalisation workshop participants

Qualitative method	Participant group	Number of participants	Gender	Placement type/Job role	Substance use
Finalisation Workshop	Professionals	14	F = 10, M = 4	Drug and Alcohol worker = 9 Social Worker = 1 Project support workers = 4	
	Young People	13 LAC = 5 Non-LAC = 8	F = 7, M = 6	Living with biological family = 6 Non-LAC Living in supported accommodation due to family breakdown due to drug use = 1 Non-LAC Living with grandparents due to family breakdown due to drug use = 1 Non-LAC Supported accommodation = 3 LAC Residential care = 1 LAC Foster care = 1 LAC.	All 13 participants were currently in/or had previously attended specialist drug and alcohol services
Total participants		27			

Within the professional workshops, participants were split into two separate groups and asked to consider either the MET or SBNT intervention and discuss what the final manuals should ‘look like’. The workshops with young people included both LAC (*n* = 5) and non-LAC (*n* = 8) whom had all experienced accessing specialist drug and alcohol treatment. The decision to include non-LAC was made as we had difficulty identifying LAC who had previously been in treatment and were still contactable. We wanted to ensure we had maximum variation of young people whom had experience of accessing drug treatment agencies to discuss the developed interventions. We held multiple workshops to enable participants to be involved in each active study site without having to travel long distances to take part. The workshops were all held within the well-established young people’s drug and alcohol services involved in the study so participants were in a familiar environment. The workshops provided an opportunity for the research team to present the preliminary findings to participants and then work collaboratively to co-produce the final manuals.

Verbatim quotes were used to identify areas of importance and to facilitate discussion between researchers and participants. Table 3 identifies the themes discussed within the workshops and the resulting recommendations for intervention development.

**Table 3:** Themes and subthemes and implication for manual adaptation

Theme	Subtheme	Implications
Therapeutic relationships	Time and reciprocal self-disclosure Genuine care Non- judgemental approach	Practitioners to make contact with LAC and to have a pre-treatment sessions to engage the YP to build up a rapport and encourage a relationship prior to commencing work. Therapists to have the option to make safe self-disclosures within the sessions.
Engagement and Challenges	The need to use creative methods to enhance engagement. YP inability to recognise support Treatment goals wider than substance use.	Resources were developed to complement each session within the MET and SBNT manuals. The resources provided a worksheet and suggested activities to work in a creative way with LAC. The original criteria for a ‘network member’ was made more flexible to enable less traditional members to engage with sessions and act as a support. Treatment goals could be wider than substance misuse alone.

Within each theme, areas of potential intervention adaptation were discussed and the findings from the qualitative interviews, focus groups and workshops have resulted in a number of adaptations being made to the manualised interventions. Participants (inclusive of professionals and LAC) made recommendations regarding the need for additional resources such as worksheets and exercises to be available to support the training of drug and alcohol workers and to complement the adapted manuals. Practitioners requested appointment cards that identified appointments were for SOLID as opposed to the usual treatment services and a pre-treatment session was requested to enable practitioners to attempt to build a rapport prior to commencing sessions.

The MET intervention originally consisted of three sessions and was increased to six to enable more time for a therapeutic relationships to be built. The SBNT interventions originally consisted of eight sessions and was reduced to six sessions, in an attempt to keep the sessions focused and in turn keep LAC interested and engaged in the work being done. However, as the interventions are delivered to meet the young person’s needs, they could be completed in less than six sessions if appropriate. If more than six sessions were required, LAC would be referred into tier 3 structured services for further work to take place.

Drug and alcohol practitioners are encouraged to consider a range of approaches within sessions including a mixture of the traditional therapeutic approach, sessions inclusive of creative writing, arts and crafts and/or the completion of more formal worksheets. Allowing a flexible approach to be used in the interventions is hoped to result in LAC engaging with practitioners using a method they feel comfortable with.

Following the workshops, thematic analysis of the workshop data took place. The qualitative data collected across the different stages of the formative work were analysed separately in the first instance. However, the data from the initial set of qualitative interviews and the workshop data were also considered in conjunction with each other to establish where findings from each stage converged, offered complimentary information on the same issue and where any data appeared to contradict each other. Once analysis had been completed the final adaptation of both manuals took place. To ensure rigour within the analysis process, a number of strategies were used. We used the professional workshops as a forum to member check our data, 10 of the professional participants were respondents from whom the data were originally obtained. The data were presented at study management meeting to allow colleagues to discuss the data and ongoing communication took place between the on the ground researchers developing the manuals and the intervention authors Professor Alex Copello (SBNT) and Dr. Gillian Tober (who has adapted both MET and SBNT for other clinical trials) to ensure the core components of each approach were retained throughout the adaptation process.

## Discussion/results

The current paper gives a step by step guide, based on the Medical Research Council (MRC) framework, of how to develop and adapt complex interventions [18] using the SOLID study as a case example. The paper focused specifically on the *process* of the interventional development and highlights how the interventions were adapted to reflect the practicalities of working with a LAC population, often presenting with complex needs. The formative work explored multiple contextual components associated with being involved in the care system and participants discussed issues such as stigma, power and lack of control. As far as possible, the manual addressed these issues, specifically revising the training and manuals to focus on the issues of practitioners building trust, providing genuine care, being non-judgemental, working creatively and having treatment goals wider than substance misuse. Whilst it is outside of this *process* paper to explore the contextual components in depth, a linked paper focusing on the ‘conceptualisation of care’ goes some way to acknowledging the important components of care (Brown R, Alderson H, Kaner E, McGovern R, Lingam R. "There are carers, and then there are carers who actually care" Conceptualizations of care among looked after children and care leaver, social workers and carers. Submitted).

### Key adaptations

Key adaptations made were: the need to focus upon overcoming insecure attachments and mistrust that LAC have experienced by default of being involved in the care system and the need to be more flexible with social network members due to the fragmented nature of available support networks and repeated broken relationships. In a practical sense the need to use creative non-traditional methods to engage LAC who are known to have lower educational achievements than their peers and to overcome their repeated exposed to new workers and services due to moving into new placements. Finally, the need for treatment goals to be wider than substance misuse alone to accommodate the diverse needs of LAC whom are known to have higher rates of co-morbid mental health problems and higher levels of risk-taking behaviours such as drug and alcohol misuse. It was essential that a LAC’s needs should be addressed holistically as substance misuse rarely happens in isolation but often interacts with a wider set of problems.

Trust was a recurring theme on behalf of LAC and professionals. Many studies have shown that maintaining and developing positive relationships are highly important to LAC [22–25]. Young people in our study reported wanting to build relationships slowly, supporting McLeod’s (2010) argument that young people need time to get to know their workers before they feel comfortable sharing their personal thoughts and feelings [26]. One likely explanation for this is the likelihood of a LAC experiencing disorganised attachment patterns due to having previous violations of trust such as rejection from their birth family, repeated broken relationships, broken promises and being let down in the past [27, 28]. The SBNT approach within this study attempted to establish a broader support network that would facilitate the emergence of positive relationships that could continue beyond the intervention delivery. In effect the supportive therapeutic relationship was between the young person and their trusted adult, this relationship was facilitated by the drug and alcohol worker. This is the key difference between SBNT and MET. This approach would help to negate against any unintended consequences that may occur when the treatment episode finishes and the practitioner element of the care relationship ends. To facilitate and encourage trust between the practitioner and the LAC it was suggested that practitioners could make ‘safe self-disclosures’ within sessions. Furthermore, a pre-assessment session was written into the manual to enable practitioners to contact the young person and have an informal ‘chat’ with them prior to commencing any sessions. The pre-assessment session allowed the practitioner to discuss with the young person their preferred way to engage inclusive of preferred times, days, methods of engagement and to arrange their first appointment. For both the MET and SBNT approaches, It is important to consider the ‘ethic of care’ concept. Practitioners need to understand the relational aspects present within the lives of LAC as the nature of care is complex and in fact LAC often define care by its absence [29]. There is a need to understand that care may be experienced by LAC as oppressive as for many young people the receipt of statutory care is involuntary [30].

The current qualitative study highlighted the importance of LAC feeling respected and having their voices heard. This translated within the workshops to a co-produced intervention wherein the LAC sets the agenda and pace of work. This is a common finding of research into young people’s experience of service usage [23, 26] and LAC in particular have been found to want choice, control and more involvement in decision making [27, 29, 31], perhaps because they have lacked control in other areas of their lives. It was therefore recommended that practitioners work in a person centred way to attempt to address the stated needs of LAC.

In response to feedback, hand-outs and worksheets were developed to complement each session and the manual identified how activities could be used to keep LAC engaged both within and between sessions. Due to LAC having lower levels of functional literacy, the use of creative/visual approaches enables LAC to express their thoughts and felling more easily [32, 33]. The training with practitioners acknowledged that traditional counselling style sessions may be abandoned to opt for a more informal setting such as a coffee shop or fast food restaurant as long as confidentiality could be upheld***.*** Approaches such as Multisystemic therapy (MST), Multidimensional family therapy (MDFT) and Functional family therapy (FFT) have shown that the ability to be flexible regarding the time, place and frequency of client contact is crucial to improving engagement and retention [12, 34, 35].

The manual and training were adapted to acknowledge that LAC may struggle to adhere to one of the traditional criteria of a ‘network member’, that of a network member being ineligible if they ‘have an alcohol or drug misuse problem themselves’. This may be problematic as it is known that 25% of LAC in foster care and 42% of LAC in residential care drank alcohol at least once a month [2] 32% smoked marijuana daily [3] and 11.3% of LAC aged 16–19 years had a diagnosed substance use problem [36]. This alongside placement instability and multiple broken relationships makes maintaining positive support networks problematic [29, 37] which as identified above reinforces the benefits of delivering the adapted SBNT intervention. Additionally, the limited ability of LAC to identify sources of support, even when support was actually available, reinforced what the adapted SBNT intervention was trying to achieve, that of helping LAC to identify sources of support currently within their life.

The manual still identifies an ideal network member however the training discussed scenarios when the criteria can be amended to enable LAC to bring a supportive individual to sessions. The manual emphasised the importance for LAC to sustain engagement by widening the reach of the intervention beyond the traditional family. This was significant as young people presenting with substance use problems often come from disrupted families and traditional systemic family approaches may be problematic to deliver in such situations [38]. The manual stressed that the network could be extended to include supportive peers, or other figures perceived as being important by the LAC including e.g. teachers, social workers or possibly wider family members such as grandparents, all of the latter with potential to provide sustained and more secure attachments. The ability of support network to be inclusive of broader and more informal networks is important, especially for LAC who receive primary care outside of the more ‘traditional caregivers’ [29].

Finally, both interventions reinforced the recognition for practitioners to be flexible and responsive to the young person’s needs by designing the manuals in such a way that topics did not have to run in a set order, practitioners could organise sessions and deliver the content in a way best suited to the young person. Regarding SBNT, the original approach of having core and elective topics is designed to allow a flexible approach to be taken, this was emphasised when developing the manual for LAC. Literature identifies that it is crucial to ensure that interventions address issues of concern for the young person rather than following a strict plan that does not address their needs [34]. Additionally, **t**he manuals enabled practitioners to have the flexibility to focus on the young person’s identified goal regardless of whether it was specifically to address their substance misuse. It was essential that a young person’s needs should be addressed holistically as substance misuse rarely happens in isolation but it often interacts with wider problems [39, 40]. The rationale for this decision was that if other areas of concern for the young person were addressed it may by default impact positively upon their levels of substance use.

## Conclusion

To our knowledge this is the first study to describe the process of intervention adaptation with LAC, carers, drug and alcohol workers and social workers to refine and develop two evidence based behaviour change interventions. Overall findings highlight the importance of co-production with this group of young people and suggested that whilst original components of both interventions were feasible to deliver and acceptable, specific adaptations had to take place. Key areas included increased emphasis upon therapeutic relationships, the benefits of using creative non-traditional methods of engagement and identification of treatment goals wider than those narrowly focused on substance misuse. This paper provides an example of using the MRC framework to collect multiple perspectives to refine an intervention to decrease drug and alcohol use and support mental health of Looked after Children in the UK at scale.

## Abbreviations

BDI: Behaviour Determinant Intervention
FFT: Functional family therapy
ITEP: International Treatment Effectiveness Programme
LAC: Looked after children and care leavers
MDFT: Multidimensional family therapy
MET: Motivational Enhancement Therapy
MI: Motivational Interviewing
MRC: Medical Research Council
MST: Multi systemic therapy
NICE: National Institute for Health and Care Excellence
PPI: Public and Patient Involvement
PR: Parental Responsibility
SBNT: Social Behaviour and Network Therapy
SOLID: Supporting Looked After Children and Care Leavers In Decreasing Drugs, and Alcohol)
UK: United Kingdom
YSBNT: Youth Social Behaviour and Network Therapy
